# Is Receptor-Interacting Protein Kinase 3 a Viable Therapeutic Target for *Mycobacterium tuberculosis* Infection?

**DOI:** 10.3389/fimmu.2018.01178

**Published:** 2018-05-28

**Authors:** Michael D. Stutz, Samar Ojaimi, Gregor Ebert, Marc Pellegrini

**Affiliations:** ^1^Infection and Immunity Division, The Walter and Eliza Hall Institute of Medical Research, Melbourne, VIC, Australia; ^2^Department of Medical Biology, The University of Melbourne, Melbourne, VIC, Australia

**Keywords:** receptor-interacting protein kinase 3, tuberculosis, macrophage, necrosis, host-directed therapy, inflammation

## Abstract

The dwindling list of antimicrobial agents exhibiting broad efficacy against clinical strains of *Mycobacterium tuberculosis* (Mtb) has forced the medical community to redefine current approaches to the treatment of tuberculosis (TB). Host receptor-interacting protein kinase 3 (RIPK3) has been flagged recently as a potential target, given that it is believed to regulate necroptosis-independent signaling pathways, which have been implicated in exacerbating several inflammatory conditions and which reportedly play a role in the necrosis of Mtb-infected macrophages. To examine the therapeutic potential of inhibiting RIPK3, we infected RIPK3-deficient mice with aerosolized Mtb. We found that the loss of RIPK3 did not alter overall disease outcomes, with deficient animals harboring similar bacterial numbers in the lungs and spleens compared to their wild-type counterparts. Mtb-infected macrophages were not rescued from dying by *Ripk3* deletion, nor did this affect production of the pro-inflammatory cytokine IL-1β, both *in vitro* and *in vivo*. Infiltration of immune cells into the lungs, as well as the activation of adaptive immunity, similarly was not overtly affected by the loss of RIPK3 signaling. Collectively, our data argue against a role of RIPK3 in mediating pathological inflammation or macrophage necrosis during Mtb disease pathogenesis and thus suggest that this host protein is unlikely to be an attractive therapeutic target for TB.

## Introduction

The alarmingly rapid emergence of highly drug-resistant strains of *Mycobacterium tuberculosis* (Mtb) has given traction to therapeutic approaches that target host cellular processes rather than the pathogen itself. Investigations of these so-called host-directed therapies have largely focused on modulating the function of the macrophage to Mtb infection, given that this innate immune player is a principal host cell involved in the immunological response to tuberculosis (TB).

The receptor-interacting protein kinase (RIPK) family comprises seven members whose defining feature is a homologous serine/threonine kinase domain, but which differ widely in terms of functional domains (1). As their name implies, the RIPK proteins play fundamental roles in the response to intracellular and extracellular stimuli and have emerged as critical regulators of inflammatory and cell death signaling pathways, leading to intense interest in pharmacologically targeting these proteins (1–3). Although the functions of RIPK4-7 are poorly understood, RIPK2 is known to be required for signaling following detection of bacterial peptidoglycan derivatives by the NOD2 cytosolic receptor (3). Studies of RIPK1 have elucidated its pivotal role in dictating the outcome of death receptor signaling, in terms of whether a cell triggers pro-inflammatory gene expression or engages the apoptotic cell death pathway. However, RIPK1 also interacts with RIPK3, the best characterized role of which is the phosphorylation of mixed lineage kinase domain-like (MLKL)—a dedicated pseudokinase that is essential for the execution of a regulated necrotic form of cell death, termed necroptosis (4, 5).

We recently demonstrated that Mtb-infected human and mouse macrophages do not undergo necroptosis and that this form of cell death does not contribute to Mtb disease pathogenesis *in vivo* (6). However, phenotypic differences between *Ripk3^−/−^* and *Mlkl^−/−^* mice (both of which are unable to engage necroptosis) in numerous disease models have made it clear that RIPK3 participates in additional pathways beyond inducing necroptosis (7). Several reports have now described MLKL-independent roles of RIPK3 in pro-inflammatory signaling in macrophages and dendritic cells, including NLRP3 inflammasome activation and IL-1β maturation (8–13), and more recently, the production of type I IFN (14, 15). Such pathways reportedly contribute to protection against certain infections such as influenza A virus, with *Ripk3^−/−^* mice experiencing a heightened susceptibility to infection due to an inability of macrophages to produce type I IFN in the lungs (10, 14). The cell death-independent functions of RIPK3 have also been implicated in the pathogenesis of several diseases. For example, *Ripk3* deletion was found to attenuate disease in mouse models of kidney ischemia–reperfusion injury, myocardial infarction, and systemic inflammation (7). RIPK3-dependent inflammasome activation and IL-1β secretion by macrophages were also shown to be responsible for retinal detachment-induced photoreceptor death (16). Type I IFNs are generally associated with TB disease progression (17–19). Although IL-1β is essential for the control of Mtb (20, 21), and the NLRP3 inflammasome appears to be dispensable for its production (22), individuals carrying a polymorphism in the *IL1B* promoter region that increases IL-1β expression are more susceptible to severe active TB disease with extrapulmonary lesions (23). Intriguingly, two groups recently reported that RIPK3 promotes necrosis of Mtb-infected macrophages *in vitro* by stimulating the production of mitochondrial reactive oxygen species (ROS), *via* a pathway requiring the anti-apoptotic protein BCL-XL (24, 25). These experiments, which largely used RNAi and human and immortalized murine macrophages, were complemented with *in vivo* studies, in which *Ripk3*-deficient mice were shown to harbor fewer bacteria in the lungs following high-dose intravenous Mtb infection, although this was substantially less striking when mice were infected by low-dose aerosol (25). The authors concluded that RIPK3 signaling is thus pathogenic during Mtb infection by promoting the escape of mycobacteria from necrotic macrophages and facilitating dissemination, leading to worse disease outcomes.

Reports that RIPK3-dependent inflammatory signaling by macrophages is pathological in many infectious and non-infections disease models, and recent work concluding that RIPK3 mediates a necrotic macrophage death modality, have emphasized the potential therapeutic benefit of targeting this host protein for the treatment of TB. The development of small molecule inhibitors of RIPK3 is being aggressively pursued (26, 27) with several such compounds undergoing testing in preclinical models of disease. It is therefore of utmost importance to clearly define the involvement of RIPK3 in the pathogenesis of Mtb infection. Our previous work demonstrating that the MLKL-dependent necroptosis does not contribute to Mtb disease has enabled us to specifically examine the non-necroptotic functions of RIPK3 by using mice genetically targeted for this gene.

## Materials and Methods

### Mice

The Walter and Eliza Hall Institute of Medical Research Animal Ethics Committee reviewed and approved all animal experiments. Six- to 10-week old male and female C57BL/6, *Ripk3^−/−^* (28) (Vishva Dixit, Genentech), and *Bcl-x_L_^flox/flox^;LysM-Cre* mice (29) (James Vince, WEHI) were used and were age- and sex-matched in all experiments. Mice infected with Mtb were housed in individually ventilated microisolator cages.

### Bacteria

*Mycobacterium tuberculosis* strain H37Rv and a strain of H37Rv episomally expressing mCherry were both sourced from Nicholas P West (University of Queensland). Mycobacteria were cultured in albumin–dextrose–catalase supplemented 7H9 medium (BD Biosciences, San Jose, CA, USA) and prepared as a single-cell suspension as described previously (6). Briefly, bacteria were pelleted and washed with PBS + 0.05% Tween-80 (Sigma-Aldrich), before low-speed centrifugation at 130 × *g* for 8 min to pellet aggregated bacteria. Single-cell bacteria in the supernatant were quantitated by measuring optical density (OD; 590 nm), with an OD_590_ 0.1 estimated to be 5 × 10^7^ CFU/ml. The mCherry-expressing strain was used in some experiments as indicated and was cultured in the presence of 25 µg/ml kanamycin (Sigma-Aldrich).

### BCL-XL Inhibitor

In some experiments, mice were administered 25 mg/kg of the BCL-XL-selective inhibitor A-1331852 (30) (AbbVie Inc., North Chicago, IL, USA) by daily oral gavage. Control mice received an equivalent volume of vehicle, which consisted of 60% Phosal 50 PG (Lipoid), 27.5% PEG400 (Affymetrix), 10% ethanol, and 2.5% DMSO (Ajax Finechem). Platelet counts in the blood of treated mice were determined using an ADVIA 2120 hematology analyzer (Siemens Australia New Zealand, VIC, Australia).

### Macrophage Isolation and Infection

Bone marrow-derived macrophages (BMDMs) were prepared from WT and *Ripk3^−/−^* mice as described previously (6). Differentiated BMDMs were plated at a density of 4 × 10^5^ cells per well (12-well plates) in antibiotic-free DMEM supplemented with 10% fetal bovine serum (Sigma-Aldrich) and 15% L929-conditioned medium, which was maintained for the entire duration of the experiment. Cells were treated/infected at various multiplicities of infection (MOIs) as indicated 24 h after plating by adding single-cell Mtb suspensions into the culture.

### Cell Death Analysis

Adherent and non-adherent BMDMs were collected 48 h post-infection, stained with 25 µg/ml propidium iodide (Sigma-Aldrich) and analyzed using an LSRFortessa X-20 flow cytometer (BD Biosciences).

### Western Blots

Bone marrow-derived macrophages were harvested 24 h after infection with Mtb and/or treatment with 50 ng/ml recombinant mouse IFNγ (Biolegend). Proteins were isolated using cell lysis buffer containing 1% Triton X-100 (Sigma-Aldrich), 20 mM Tris-HCl, pH 7.5, 135 mM NaCl, 1.5 mM MgCl_2_, 1 mM EGTA, 10% glycerol, EDTA-free protease inhibitor tablets (Roche), and phosphatase inhibitor tablets (Roche). Equal quantities (30 µg) of total protein were separated under denaturing and reducing conditions using 4–12% SDS-PAGE gels (Life Technologies), transferred onto nitrocellulose membranes, blocked with 5% skim milk for 1 h, and detected using the following primary antibodies: rabbit anti-iNOS (cat. #ab178945; Abcam), rabbit anti-RIP3 (cat. #2283; ProSci; RRID: AB_203256), and rabbit anti-β-actin-HRP (cat. #5125; Cell Signaling Technology; RRID: AB_1903890). HRP-conjugated goat secondary antibodies (Southern Biotech) were applied to membranes, which were then incubated with Luminata Forte Western HRP substrate (Merck) and imaged using a ChemiDoc Touch Imaging System (Bio-Rad). Densitometry was performed using Image Lab v.5.2.1 software (Bio-Rad; RRID:SCR_014210).

### Cytokine Analysis

Bone marrow-derived macrophages were infected with Mtb in the presence of GolgiStop and GolgiPlug (BD Biosciences) for 4 h. Cells were then harvested, fixed with 4% paraformaldehyde (PFA) for 30 min, permeabilized (Perm/Wash Buffer, BD Biosciences), and stained for intracellular TNF by incubating cells with rat anti-TNF PE (clone: MP6-XT22; Biolegend; RRID: AB_315427) for 45 min at 4°C. Stained cells were analyzed by flow cytometry.

Bone marrow-derived macrophage culture supernatants were centrifuged at 500 × *g* for 5 min, filter-sterilized (0.22 µm), and analyzed for TNF and IL-1β using DuoSet ELISA kits (R&D Systems), according to the manufacturer’s instructions. Lung tissue homogenates (described below) were diluted 1:1 in cell lysis buffer containing protease inhibitors, centrifuged at 9,000 × *g* for 10 min to pellet debris, and analyzed for TNF, IL-1β, and IFNγ.

### Aerosol Infection and Quantitation of Mtb

Mice were infected with Mtb by low-dose aerosol (~100–200 CFU), using a whole-body Inhalation Exposure System (Glas-Col), as described previously (6). Three mice were sacrificed 24 h after aerosol exposure to confirm the pulmonary infection dose.

Mice were euthanized at various times post-infection by CO_2_ asphyxiation, and organs were collected and homogenized with steel beads in PBS + 0.05% Tween-80 using a Bullet Blender (Next Advance, Inc.). Homogenates were serially diluted and plated onto Middlebook 7H11 agar (BD Biosciences) supplemented with 0.5% (v/v) glycerol and 10% (v/v) OADC supplements. Colonies were counted after 21 days incubation at 37°C and were expressed as CFU/organ.

### Histology

Lungs were inflated by intratracheal perfusion with 4% PFA and fixed overnight at 4°C. Left lobes were embedded in paraffin, sectioned, and stained with hematoxylin and eosin. Slides were scanned with an Aperio ScanScope AT slide scanner (Leica Microsystems) and images analyzed using FIJI software with custom-written macros.

### Bronchoalveolar Lavage (BAL)

Mice were euthanized, and the trachea cannulated with an 18-gauge needle. BAL was performed with eight 1 ml aliquots of ice-cold Hank’s Balanced Salt Solution (Ca-/Mg-free) containing 0.5 mM EDTA. Retrieved cells were cultured in tissue culture-treated plates for 45 min to allow alveolar macrophages to adhere. Unattached cells were removed, and plates washed with PBS. Macrophages were detached with trypsin-EDTA (Sigma-Aldrich), washed with PBS, and counted using a Neubauer hemocytometer. Cells were then lysed with 0.5% Triton X-100 in PBS, serially diluted, and plated as described above to quantitate intracellular CFU.

### Immune Cell Profiling and Peptide Restimulation

Immune cells were isolated from the lungs and spleens of infected mice as described previously (6). A portion of isolated lung cells and splenocytes were stained with the following antibodies (all from BD Biosciences) for 45 min at 4°C: rat anti-CD16/CD32 (RRID: AB_394657), rat anti-CD4 BV421 (clone: GK1.5), rat anti-CD8 PE-Cy7 (clone: 53-6.7; RRID: AB_394506), rat anti-CD19 PerCP-Cy5.5 (clone: 1D3; RRID: AB_394004), rat anti-CD11b BV510 (clone: M1/70), hamster anti-CD11c APC (clone: HL3; RRID: AB_398460), rat anti-I-A/I-E (clone: 2G9; RRID: AB_394958) FITC, rat anti-Ly-6G/Ly-6C PE (clone: RB6-8C5; RRID: AB_394644), rat anti-PD1 PE (clone: J43; RRID: AB_394284), and hamster anti-CD69 BV421 (clone: H1.2F3; RRID: AB_2687478). Cells were washed and fixed with 4% PFA for 30 min.

Approximately 2 × 10^6^ splenocytes and 3 × 10^6^ lung cells were cultured in Iscove’s modified Dulbecco’s medium containing 10% FCS, β-mercaptoethanol, penicillin/streptomycin, GolgiStop, and GolgiPlug. Cells were stimulated with 5 µg/ml Mtb early secreted antigenic target 6 (ESAT-6; MTEQQWNFAGIEAAA) peptide (GenScript), and incubated for 5 h. Cells were then harvested, surface stained with rat anti-CD4 BV421 and rat anti-CD8 PE-Cy7, fixed with 4% PFA for 30 min, and permeabilized. Intracellular cytokine staining was performed by incubating cells with rat anti-TNF PE (clone: MP6-XT22; Biolegend) and rat anti-IFNγ FITC (clone: XMG1.2; BD Biosciences; RRID: AB_395375) for 45 min at 4°C. Stained cells were analyzed by flow cytometry.

### Statistics

Prism 6.0h (GraphPad Software) was used to perform statistical tests. Groups were compared by unpaired two-tailed *t* tests for parametric data. Non-parametric data were log-transformed for statistical analysis, or when this failed to normalize the data, Mann–Whitney tests were used. Holm–Sidak correction was applied for multiple *t* tests.

## Results

### Macrophage Responses to Mtb Infection Are Unaltered by the Loss of RIPK3

Macrophages are the principal host cells involved in the immune response to Mtb infection. We therefore addressed whether the loss of the RIPK3 signaling axis altered any aspects of the anti-mycobacterial response of murine macrophages.

**Figure 1:**
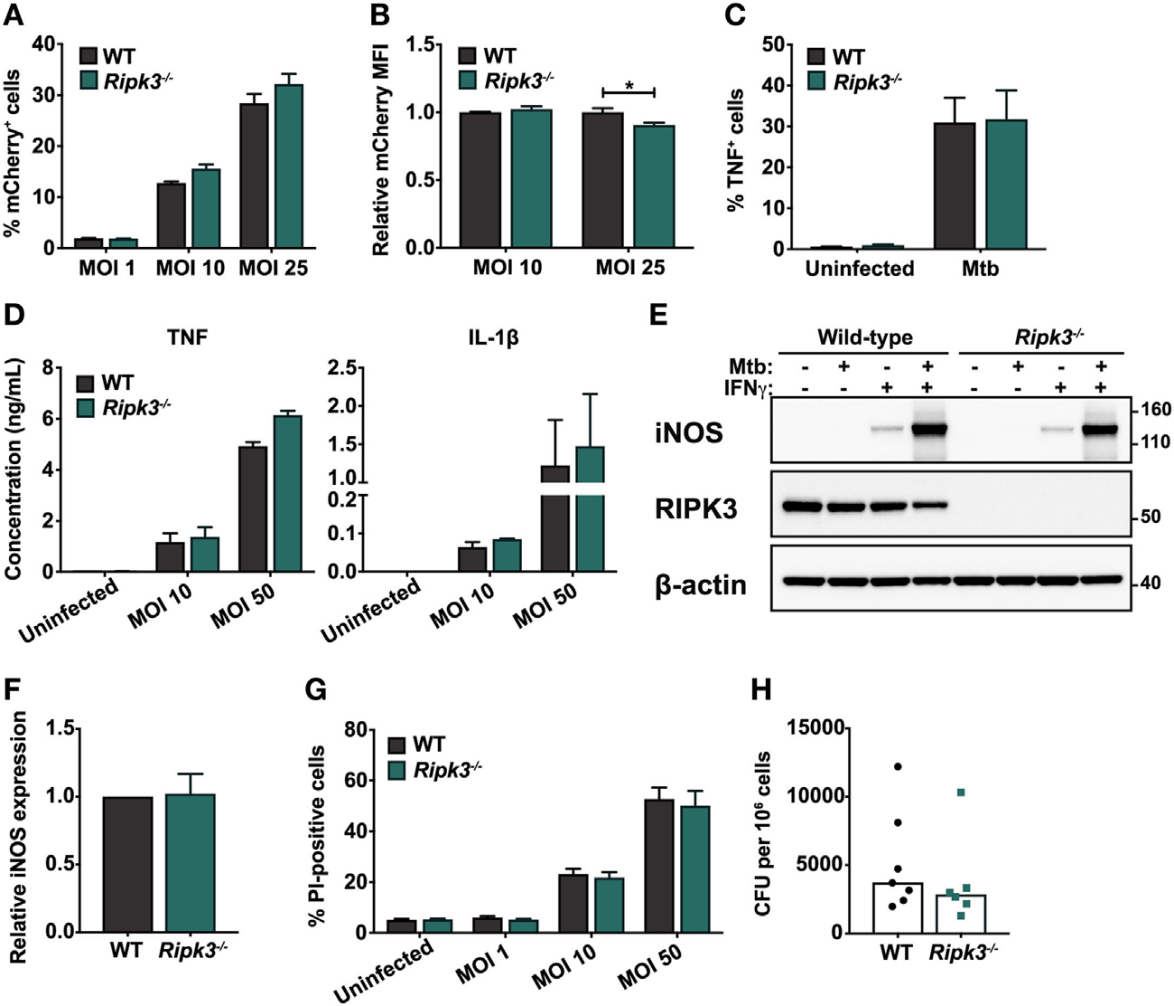
Responses of murine macrophages to *Mycobacterium tuberculosis* (Mtb) infection are unaltered by the loss of receptor-interacting protein kinase 3 (RIPK3). **(A)** Percentage of total bone marrow-derived macrophages (BMDMs) positive for mCherry fluorescence. Cells were infected with mCherry-expressing Mtb H37Rv for 4 h at the indicated multiplicities of infection (MOIs). **(B)** Relative median fluorescence intensity (MFI) of mCherry in mCherry-positive cells in **(A)**. **(C)** Percentage of total BMDMs producing TNF in response to Mtb infection. Cells were infected with Mtb for 4 h at an MOI of 25 and analyzed by intracellular cytokine staining and flow cytometry. **(D)** Concentrations of TNF and IL-1β in culture supernatants of WT and *Ripk3^−/−^* BMDMs 24 h post-infection with various MOIs of Mtb, determined by ELISA. **(E)** Western blot of inducible nitric oxide synthase (iNOS) expression in wild-type (WT) and *Ripk3^−/−^* BMDMs infected with Mtb (MOI 10) and/or treated with IFNγ (50 ng/ml). Numbers to the right of the panels represent the positions of protein size markers (in kDa). **(F)** Densitometric quantitation of relative iNOS protein expression in BMDMs infected with Mtb and primed with IFNγ (adjusted for actin loading). **(G)** Death of WT and *Ripk3^−/−^* BMDMs 48 h after infection with various MOIs of Mtb. The amount of cell death was determined by propidium iodide staining and flow cytometry. **(H)** Intracellular bacterial numbers in alveolar macrophages isolated from the bronchoalveolar lavage (BAL) of WT and *Ripk3^−/−^* mice 4 weeks post-infection. Each point represents one mouse (*n* = 6–7). Data were pooled from two **(D,H)**, three **(E,F)**, or four **(A–C,G)** independent experiments. Graphs show median **(H)** or mean ± SEM **(A–D,F–G)**. There were no statistically significant differences between genotypes (*p* > 0.05) unless indicated. *p < 0.05.

First, we considered the possibility that the loss of RIPK3 signaling may affect the ability of macrophages to internalize Mtb. Differences in the initial bacterial burden would confound interpretation of our subsequent experiments. To address this, we infected BMDMs derived from wild-type (WT) and *Ripk3^−/−^* mice with an mCherry-expressing Mtb strain and analyzed mCherry fluorescence in BMDMs by flow cytometry 4 h after infection. The proportion of cells harboring intracellular mycobacteria (mCherry fluorescence) was similar between WT and *Ripk3^−/−^* BMDMs at all MOIs (Figure 1A). The median fluorescence intensity of the mCherry signal—a marker of intracellular bacterial numbers—was comparable between genotypes, albeit marginally lower in *Ripk3^−/−^* cells infected at higher MOI (Figure 1B).

We next examined the macrophage response to intracellular Mtb, which involves the secretion of a number of cytokines including TNF and IL-1β, as well as the activation of microbial killing mechanisms such as inducible nitric oxide synthase (iNOS). These components of the immune response are indispensable during infection *in vivo* (20, 31, 32). We found that the loss of RIPK3 did not alter the proportion of BMDMs producing TNF following Mtb infection, as determined by intracellular cytokine staining (Figure 1C). Additionally, the quantities of TNF and IL-1β secreted into the tissue culture supernatant over a 24 h period following infection were comparable between genotypes (Figure 1D). We also did not observe any difference in the ability of *Ripk3^−/−^* BMDMs to express iNOS upon infection (Figures 1E,F). Despite activation of antimicrobial responses following exposure to Mtb, most infected macrophages ultimately die, and RIPK3 has been implicated as a mediator of necrotic macrophage death (24, 25). However, we found no differences between WT and *Ripk3^−/−^* BMDMs in terms of the proportion of cells dying in response to Mtb infection at various MOIs (Figure 1G).

Receptor-interacting protein kinase 3 deficiency did not result in any defects in terms of Mtb internalization or activation of antimicrobial defenses, suggesting that during infection *in vitro*, RIPK3 does not impact on the capacity of macrophages to respond to intracellular Mtb. However, a multitude of factors contribute to the macrophage response *in vivo*, which cannot be modeled *in vitro*. Thus, to determine whether RIPK3 deficiency impacted on intracellular Mtb burdens during physiological infection *in vivo*, we isolated alveolar macrophages from the lungs of Mtb-infected mice by BAL and assessed intracellular CFU by plating cell lysates. However, there was no significant difference in intracellular bacterial numbers between genotypes (Figure 1H).

### The Loss of RIPK3 Signaling Ultimately Does Not Alter Disease Outcomes *In Vivo*

**Figure 2:**
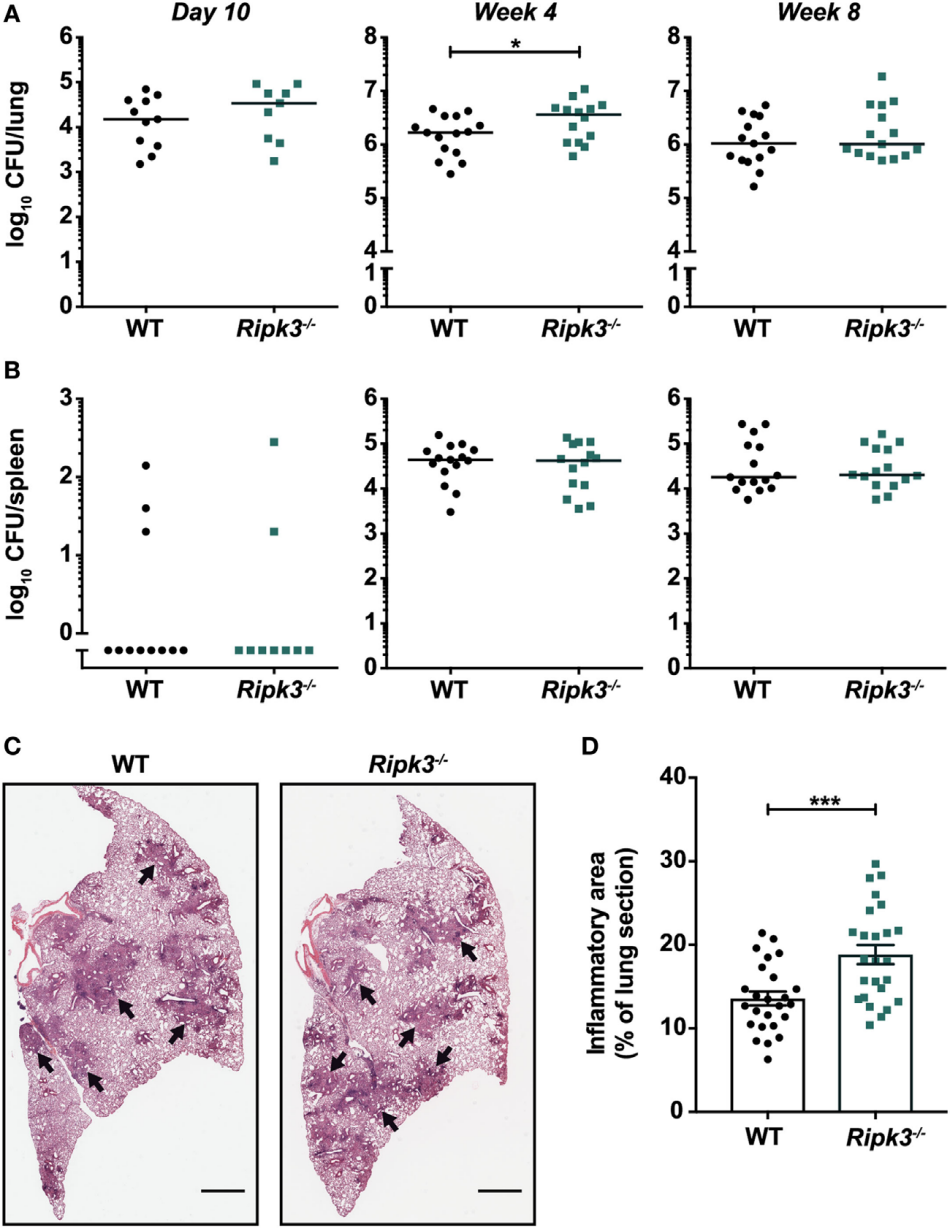
Impact of receptor-interacting protein kinase 3 deficiency on *Mycobacterium tuberculosis* (Mtb) disease pathogenesis in mice. Bacterial burden in the **(A)** lungs and **(B)** spleens of mice at various time points post-infection with aerosolized Mtb. Data were pooled from two (day 10 and week 8) or three (week 4) independent experiments (*n* = 9–15 per time point and genotype). **(C)** Lung histology of mice 4 weeks post-infection. Black arrows indicate examples of inflammatory areas. Left lobes were stained with hematoxylin and eosin. Representative of 25 mice per genotype. Scale bar represents 1 mm. **(D)** Quantitation of inflammatory areas in H&E-stained lung sections of mice 4 weeks post-infection. Data were pooled from five independent experiments (*n* = 25 per genotype). Graphs show median **(A,B)** or mean ± SEM **(D)** and each point represents one mouse. There were no statistically significant differences between genotypes (*p* > 0.05) unless indicated. *p < 0.05; ***p < 0.001.

Abrogating RIPK3 signaling in macrophages by deleting *Ripk3* did not promote anti-mycobacterial activity or prevent macrophage death *in vitro*. However, we considered the possibility that macrophage responses in the context of *Ripk3* deletion may differ in the presence of other immune cells and cytokines that are involved in infection *in vivo*. Thus, we examined whether targeting RIPK3 signaling *in vivo* promoted the clearance of Mtb by using *Ripk3^−/−^* mice and a physiologically relevant aerosol model of infection. We found that the bacterial burdens in the lungs of *Ripk3^−/−^* mice were indistinguishable from their WT counterparts 10 days post-infection (Figure 2A). Surprisingly, we noted a small increase in bacterial number 4 weeks post-infection, which correlated with a very mild increase in the size of inflammatory lesions in the lung (Figures 2A,C,D). However, this slight worsening of disease in *Ripk3^−/−^* mice at 4 weeks post-infection was not observed at a later time point (Figure 2A), with all mice surviving the entire duration of the study. Bacterial burdens in the spleen at all time points were indistinguishable between genotypes (Figure 2B).

**Figure 3:**
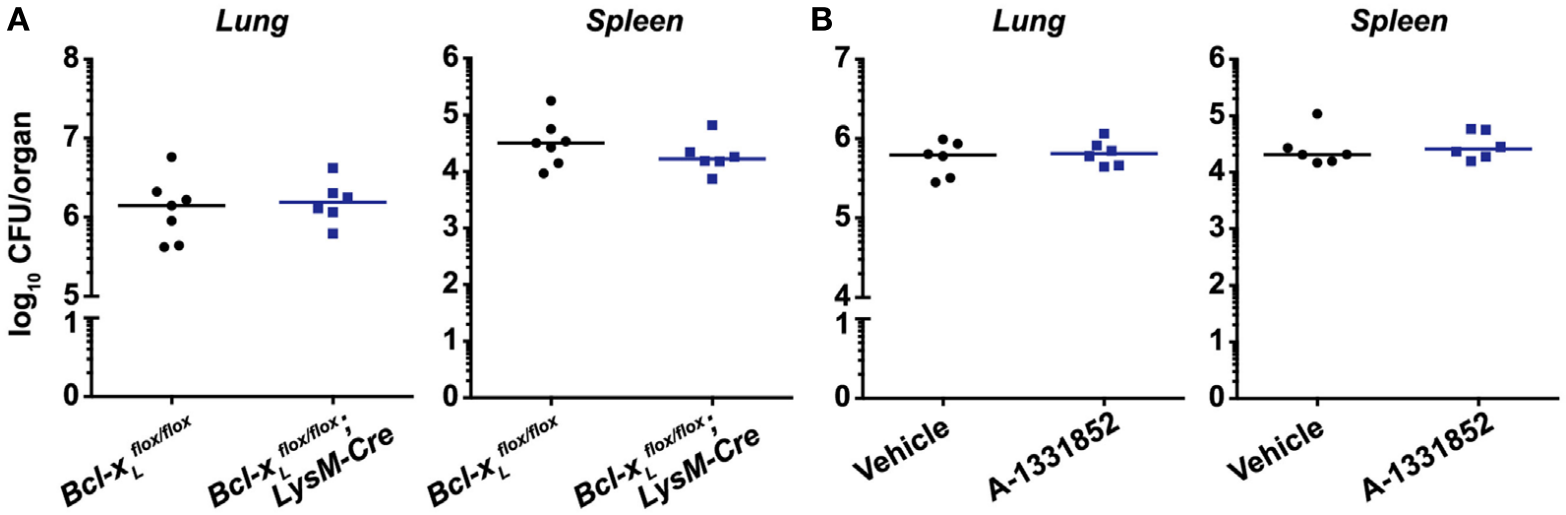
BCL-XL signaling does not contribute to *Mycobacterium tuberculosis* (Mtb) disease pathogenesis and is not a viable therapeutic target for Mtb infection. **(A)** Bacterial burden in the lungs and spleens of mice with a conditional deletion of *Bcl-x_L_* in myeloid cells 4 weeks post-infection with aerosolized Mtb (*n* = 6–7 per genotype). **(B)** Bacterial burden in the lungs and spleens of mice treated with vehicle or 25 mg/kg of the BCL-XL-selective inhibitor A-1331852 on 10 days over a 2-week period, commencing 4 weeks post-infection (*n* = 6 per treatment group). Graphs show median, and each point represents one mouse. There were no statistically significant differences between genotypes (*p* > 0.05).

Receptor-interacting protein kinase 3 was reported to promote macrophage necrosis and therefore disease progression in a process dependent on BCL-XL (25). This would imply that deletion or inhibition of BCL-XL would prevent necrosis and reduce disease severity. However, we found that bacterial burdens in the lungs and spleens of mice deficient for BCL-XL specifically in myeloid cells were indistinguishable from WT littermate controls (Figure 3A). To examine the therapeutic potential of targeting BCL-XL, which may be masked when animals are deficient in this protein from ontogeny, we used the BCL-XL-selective inhibitor A-1331852 to treat Mtb-infected mice (30). We confirmed *in vivo* pharmacologic activity of A-1331852 by demonstrating a significant reduction in platelet numbers in a cohort of naïve mice treated with the inhibitor (Figure S1 in Supplementary Material). Platelet survival depends on BCL-XL (33), and its inhibition thus results in thrombocytopenia (30), which confirms on-target BCL-XL inhibition. However, 4-week-infected mice treated with 10 doses of A-1331852 over a 2-week period again did not differ from vehicle-treated mice in terms of pulmonary and splenic Mtb bacterial numbers (Figure 3B).

### Immunological Responses to Mtb Are Unaffected by the Loss of RIPK3

**Figure 4:**
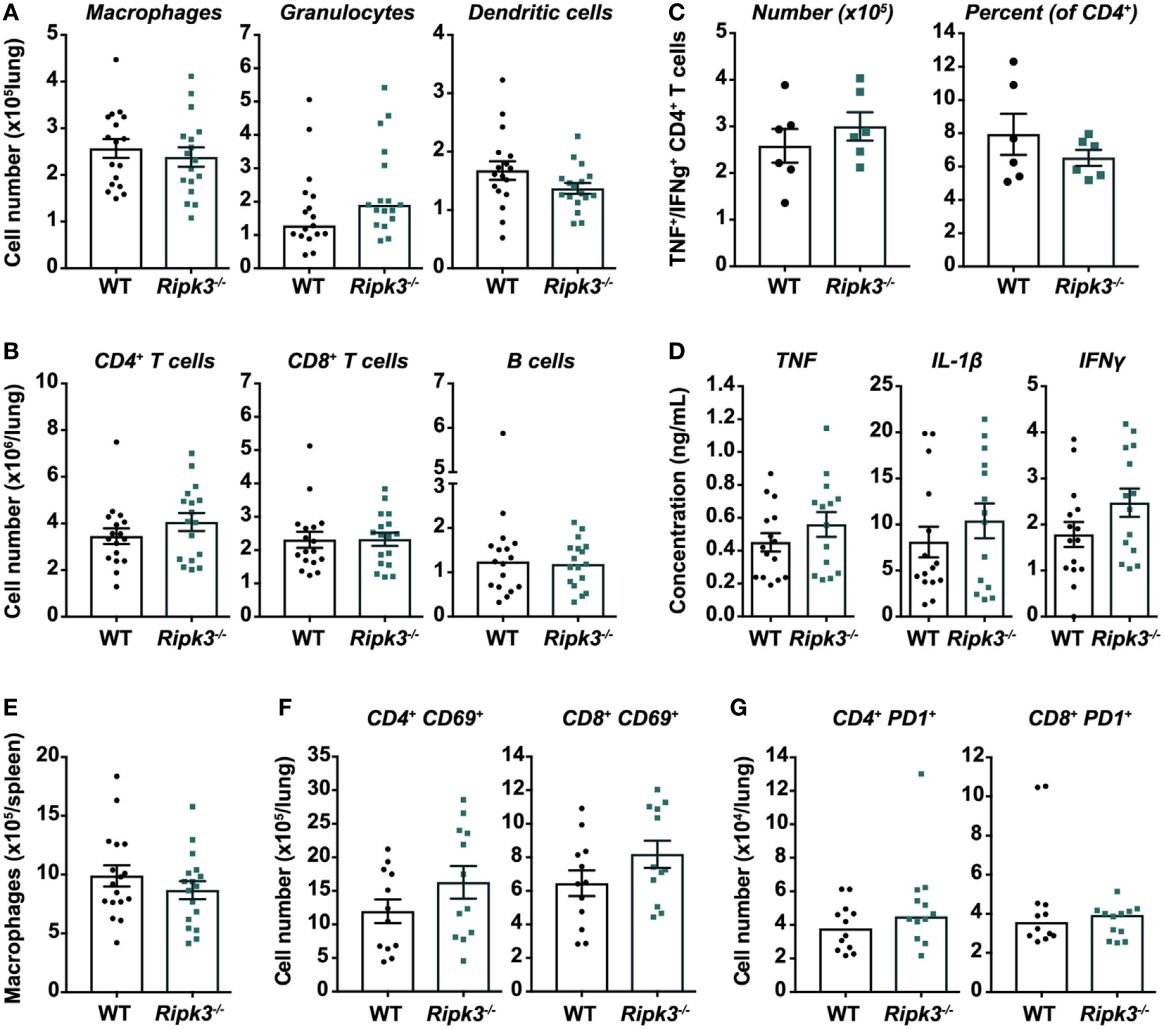
Immune responses in *Mycobacterium tuberculosis* (Mtb)-infected mice are unaltered by receptor-interacting protein kinase 3 deficiency. Total number of **(A)** myeloid and **(B)** lymphoid immune cells in the lungs of mice infected with aerosolized Mtb. Flow cytometry was used to identify macrophages/monocytes (CD11b^+^ Gr1^low^), granulocytes (CD11b^+^ Gr1^high^), dendritic cells (CD11c^+^ MHCII^+^), CD4^+^ and CD8^+^ T cells, and B cells (CD19^+^ MHCII^+^). Data were pooled from three independent experiments (*n* = 17 per genotype). **(C)** Number of Mtb early secreted antigenic target 6 (ESAT-6)-specific CD4^+^ T cells in the lungs of Mtb-infected mice. Cells were isolated from the lungs and stimulated *ex vivo* with Mtb ESAT-6 peptide, and polyfunctional (TNF^+^ IFNγ^+^) CD4^+^ T cells identified by intracellular cytokine staining and flow cytometry. Representative of two independent experiments (*n* = 6 per genotype and experiment). **(D)** Concentrations of TNF, IL-1β, and IFNγ in lung homogenates from infected mice, quantitated by ELISA. Data were pooled from three independent experiments (*n* = 14–15 per genotype). **(E)** Number of macrophages in the spleens of wild-type and *Ripk3^−/−^* mice 4 weeks post-infection with aerosolized Mtb. Data were pooled from three independent experiments (*n* = 17 per genotype). Number of **(F)** acutely (CD69^+^) and **(G)** chronically (PD1^+^) activated CD4^+^ and CD8^+^ T cells in the lungs of infected mice. Data were pooled from two independent experiments (*n* = 12 per genotype). All analyses **(A–G)** were performed at 4 weeks post-infection with aerosolized Mtb. Graphs show median or mean ± SEM, and each point represents one mouse. There were no statistically significant differences between genotypes (*p* > 0.05).

Although we did not observe any improvement in Mtb disease outcomes by deleting *Ripk3*, it remained plausible that any benefit conferred to macrophages by the loss of RIPK3, as the literature would suggest, was nullified by detrimental changes in the function of other immune cells. We thus examined whether there were any differences in immunological responses to Mtb infection in *Ripk3^−/−^* versus WT mice at 4 weeks post-infection. First, the number of macrophages in the lungs and spleens was similar between genotypes (Figures 4A,E). Overall, there were also no statistically significant differences in the number of other innate or adaptive immune cells including granulocytes, dendritic cells, T cells, and B cells (Figures 4A,B), or in the number or percentage of Mtb ESAT-6-specific (IFNγ/TNF-producing) CD4^+^ T cells in the lungs of infected mice (Figure 4C). Consistent with these results, the concentrations of TNF, IL-1β, and IFNγ in lung homogenates were comparable between genotypes (Figure 4D).

However, although there were no statistically significant differences in immune/inflammatory markers, we did observe slight trends toward increased number of granulocytes, CD4^+^ T cells, and acutely activated (CD69^+^) CD4^+^ and CD8^+^ T cells in the lungs of *Ripk3^−/−^* mice (Figures 4A,B,F,G).

## Discussion

The identification of necroptosis-independent roles of RIPK3 in driving counterproductive inflammatory responses, as well as very recent reports of a role for this protein in the necrosis of Mtb-infected macrophages, has led to the suggestion that RIPK3 may represent a therapeutic target for TB. However, our data challenge this notion and show convincingly that targeting host RIPK3 does not represent a viable therapeutic option for Mtb infection.

Using a low-dose aerosol model of Mtb infection of mice, which mimics the natural acquisition of this pathogen by humans, we showed that the loss of *Ripk3* did not fundamentally alter disease outcomes in Mtb-infected mice. Our results were somewhat surprising in light of recent reports suggesting that RIPK3 signaling is pathogenic during Mtb infection due to its induction of a necrotic form of macrophage death, which promotes bacterial dissemination (24, 25). The authors described a 0.5 log decrease in bacterial numbers in the lungs of *Ripk3^−/−^* mice 4 weeks post-infection, in a high-dose intravenous challenge model (25). It is unclear, however, whether this phenotype persisted at later time points. Interestingly, when a more physiological low-dose aerosol infection model was used, akin to that in the present study, the difference appeared much less marked, more closely reflecting our results. The discrepancy between the intravenous and aerosol models, in terms of the robustness of the difference in bacterial numbers, is intriguing and may reflect the consequences of systemic immune activation arising from intravenous exposure to high doses of Mtb, which may be mitigated to a degree by *Ripk3* deletion. Macrophage necrosis induced by RIPK3 was postulated to require the anti-apoptotic protein BCL-XL (25). This could represent an attractive therapeutic target, since several compounds that inhibit this protein are in clinical or pre-clinical trials (30, 34). However, perhaps not surprisingly, given the absence of a phenotype in *Ripk3^−/−^* mice, we found that neither the conditional deletion of *Bcl-x_L_* in myeloid cells nor the pharmacologic inhibition of BCL-XL after infection imparted any benefit to mice in terms of reduced bacterial burden. Overall, our data do not support a role for *Ripk3* in mediating macrophage death, *via* necrosis or other forms of cell death. *Ripk3* deficiency did not protect mouse macrophages from dying following Mtb infection *in vitro*, and we observed no difference in macrophage numbers in the lungs or spleens of infected WT and *Ripk3^−/−^* mice.

Previous studies addressing RIPK3 function during Mtb infection used either *Ripk3^−/−^* BMDMs (as we have done) or RNAi knockdown of immortalized mouse macrophages (J774A.1) or human monocyte-derived macrophages, all of which were reportedly protected to varying degrees from Mtb-induced death by the loss of RIPK3 (24, 25). The source of this discrepancy between our work and these previous studies is unclear. A possible explanation is species differences between human and mouse cells, but this seems unlikely given that one of these studies showed the same phenotype in human macrophages as in mouse BMDMs (25). Although the time point for death analysis was consistent (48 h for mouse macrophages), different MOIs (and potentially means of determining MOI) and methods of quantitating cell death were used, and one study used a different strain of Mtb (24). Nonetheless, we examined death at several MOIs that covered the range used in these previous studies, as well as a validated and sensitive assay for cell death. Zhao et al. (25) correlated the reduced necrotic death of RIPK3-deficient cells with lower intracellular Mtb growth after several days of *in vitro* culture. Our work showed that RIPK3 did not impact the ability of BMDMs to phagocytose Mtb upon initial exposure. We also assessed intracellular replication of Mtb, but to take into account the totality of factors that impact on bacterial replication *in vivo* (including other immune cells and cytokines), we performed this analysis on macrophages isolated from the BAL of infected mice. However, we found no difference between genotypes. This is consistent with the absence of any change in the expression of iNOS in *Ripk3^−/−^* cells, which has been shown to be a major factor restricting Mtb growth in mice (31). This would suggest that any difference in intracellular bacterial numbers *in vitro* does not translate to infection *in vivo*. This prior study also reported that RIPK3 promotes ROS production by macrophages, and this was essential to the proposed mechanism for RIPK3-mediated necrosis. We did not examine ROS production as we observed no disparities in cell death or intracellular Mtb burdens between WT and *Ripk3^−/−^* macrophages, nor any difference in disease outcomes between these genotypes of mice. Our *in vivo* data, which defines the overall response in the context of all contributors to disease pathogenesis, would suggest that any differences in ROS generation between these two genotypes are mitigated on both a cellular and organism level, such that they do not impact overall disease outcomes.

Although most disease models have identified RIPK3 as a mediator of pathological inflammation, others, including models of influenza A and West Nile virus infection, have highlighted situations in which RIPK3 signaling in fact promotes immunity (14, 35). Indeed, we noted a very marginal increase in bacterial numbers and inflammation 4 weeks post-infection with Mtb in mice lacking RIPK3. However, this difference appeared to be transient and was not observed at any other time point, including later stages of infection. Mice begin to control Mtb replication around 3–4 weeks post-infection, coinciding with the development of adaptive immunity (36, 37). We speculate that RIPK3 may thus play a small, transient role in regulating protective immunity during the handover between the early innate and the adaptive phases of the immune response. Nevertheless, the increase in bacterial number in *Ripk3^−/−^* mice was not borne out of any defect in IL-1β or TNF production in the lungs. The secretion of certain pro-inflammatory cytokines including IL-1β by macrophages and dendritic cells can be induced by RIPK3 under certain conditions, with *Ripk3* deletion attenuating inflammasome activation and IL-1β processing following LPS stimulation or infection with certain RNA viruses (9–11). However, we did not observe any defect in IL-1β production by *Ripk3^−/−^* macrophages infected with Mtb, consistent with our *in vivo* data. This may be due to the fact that the cellular inhibitor of apoptosis and X-linked inhibitor of apoptosis proteins restrict inflammasome activation by RIPK3 and must themselves be inhibited/deleted to liberate this aspect of RIPK3 signaling (8, 9, 11, 12). It is thus unclear why *Ripk3^−/−^* mice had a mild and transient impairment in Mtb control at 4 weeks post-infection. However, RIPK3 interacts with other signaling molecules including RIPK1, and this interaction has been shown to regulate multiple signaling pathways such as ERK, cFos, and NF-κB (13), and potentially JNK (38), leading to changes in inflammatory responses. In fact, RIPK3 has been implicated in the production of several cytokines in addition to IL-1β during host responses to pathogens, including type I IFN (14, 15) and chemokines (35), from immune and non-immune cell types, respectively. We hypothesize that perhaps deficiencies in such cytokines in *Ripk3^−/−^* mice at the critical interphase between innate and adaptive immunity may have conspired to produce the observed phenotype.

Collectively, our findings indicate that in the context of Mtb infection, RIPK3 does not play a fundamental role in regulating inflammatory responses or necrotic macrophage death *in vivo*, at least not in a non-redundant capacity. We would, therefore, argue against the pursuit of RIPK3 inhibition as a tractable therapeutic option for TB.

## Ethics Statement

The Walter and Eliza Hall Institute of Medical Research Animal Ethics Committee reviewed and approved all animal experiments.

## Author Contributions

MS, SO, and MP conceived and designed the research. MS, SO, and GE performed the experiments and analyzed the data. MS and MP wrote the manuscript. All authors contributed to manuscript revision, read, and approved the submitted version.

## Conflict of Interest Statement

The authors declare that the research was conducted in the absence of any commercial or financial relationships that could be construed as a potential conflict of interest.
