# Supplementary material for: Is Receptor-Interacting Protein Kinase 3 a Viable Therapeutic Target for Mycobacterium tuberculosis Infection?
Source: Front Immunol. 2018 May 28;9:1178. doi: 10.3389/fimmu.2018.01178 (PMC5985376; doi:10.3389/fimmu.2018.01178)
Supplement: Supplementary file 1 [file presentation_1.PDF]

Supplementary Material

Is RIPK3 a Viable Therapeutic Target for *Mycobacterium tuberculosis* Infection?

Michael D Stutz, Samar Ojaimi, Gregor Ebert, Marc Pellegrini*

*** Correspondence:** Marc Pellegrini, pellegrini@wehi.edu.au

SUPPLEMENTARY FIGURES

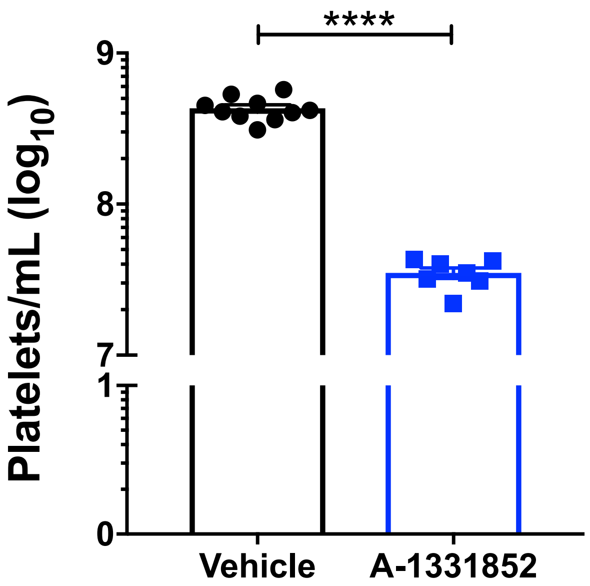

Supplementary Figure S1: *In vivo* pharmacologic activity of the BCL-XL-selective inhibitor A-1331852. Naïve mice were treated with 25 mg/kg A-1331852 or vehicle daily for eight consecutive days, and whole blood from cardiac bleeds analyzed for platelet concentration at the completion of treatment (n = 7-10 per treatment group). Graph shows mean ± SEM and each point represents one mouse. **** $p < 0.0001$.
